# Obtaining an animal welfare status in Norwegian dairy herds—A mountain to climb

**DOI:** 10.3389/fvets.2023.1125860

**Published:** 2023-02-24

**Authors:** Conor Barry, Kristian Ellingsen-Dalskau, Randi Therese Garmo, Stine Grønmo Kischel, Christoph Winckler, Camilla Kielland

**Affiliations:** ^1^Department of Production Animal Clinical Sciences, Faculty of Veterinary Medicine, Norwegian University of Life Sciences, Ås, Norway; ^2^Department for Animal Health, Animal Welfare and Food Safety, Norwegian Veterinary Institute, Ås, Norway; ^3^TINE SA, Oslo, Norway; ^4^Department of Sustainable Agricultural Systems, Institute of Livestock Sciences, University of Natural Resources and Life Sciences, Vienna, Austria

**Keywords:** animal welfare, Welfare Quality^®^, dairy cattle, loose-housed, regional variation, Scandinavia, prevalence, free-stall housing

## Abstract

**Introduction:**

Knowing the national status of animal welfare, one can identify welfare problems and set a benchmark against which improvements can be compared. Such a status is potentially invaluable for tangible, sustained animal welfare improvement. The objective of this cross-sectional study was to report the status of animal welfare in Norwegian loose-housed dairy herds as assessed using the Welfare Quality^®^ Assessment Protocol. Additionally, we investigated if the welfare status varied on a regional basis.

**Methods:**

In total, 155 herds in eight of Norway's eleven counties were assessed by six trained Welfare Quality^®^ assessors. This article presents the herd prevalences of common welfare issues in dairy production in Norway, as well as integrated welfare scores. To determine whether welfare status varied regionally in Norway, generalized linear modeling was used to estimate the mean welfare score for five regions in the four Welfare Quality^®^ principles: A. Good feeding, B. Good housing, C. Good health, and D. Appropriate behavior. These estimated mean welfare scores and their 95% confidence intervals were subsequently assessed for significant variation.

**Results:**

Encouraging findings included the low mean herd prevalence of ‘very lean' cows (3.0%) and the high proportion of cows (59.8%) which could be touched during avoidance distance testing, indicating a positive relationship between stockpeople and their cattle. Challenges affecting the welfare of Norwegian dairy cows were also identified. Of particular concern were issues related to the cows' environment such as prolonged times needed to complete lying down movements and integument alterations. No herd was completely free of changes to the integument and, on average, 77.9% of each herd were affected either mildly or severely. Animal welfare did not appear to vary much between the five regions assessed. Our investigation revealed significant regional variation between two regions (Trøndelag and Vestlandet North) in only the Welfare Quality^®^ principle Good housing (*p* < 0.01).

**Discussion:**

The almost complete absence of regional variation demonstrates that animal welfare status generally varies most at herd level. In conclusion, both welfare challenges and encouraging findings were identified in loose-housed Norwegian dairy herds. To improve animal welfare, herd-specific interventions are most likely to be effective in these herds.

## 1. Introduction

Obtaining a welfare status at a national level is challenging, a mountain to climb, so to speak. With a national status, one can identify welfare problems and set a benchmark against which future improvements can be compared, both on a national and herd level. A status of animal welfare is potentially invaluable for tangible, sustained animal welfare improvement. The objective of this cross-sectional study was to report the status of animal welfare in Norwegian loose-housed dairy herds as assessed using the Welfare Quality^®^ (WQ^®^) Assessment Protocol for dairy cows (1). Our aim was to report these integrated WQ^®^ scores and the underlying observed prevalences of common welfare issues in dairy production in Norway. Additionally, we investigated if the welfare status varied on a regional basis.

Animal welfare is high on the political and societal agenda for many countries (2). European citizens are becoming increasingly concerned that food production systems and other activities such as transport should be sustainable. Animal welfare is an important aspect of sustainability and product quality. Negative perceptions may result in consumers refusing to buy products (3). The future of the dairy industry may therefore be dependent on consumers' confidence that the cows producing the milk in their dairy products are treated appropriately. Norway is no exception. The parliamentary white paper specifically aiming to improve animal welfare in production animals announced by the Norwegian government at the end of 2021 is evidence of this (4).

Concern about animal welfare is nothing new. Stockpeople have always been concerned about the condition of animals in their care and have tried to ensure that they are healthy and well nourished. Increasingly, this well-being is seen as more than just the absence of illness or injury (5). Instead, the focus is shifting toward what was described by the Farm Animal Welfare Council as “a life worth living”. The well-established WQ^®^ protocol, for example, includes measures of positive as well as negative welfare. Furthermore, WQ^®^ follows the recommendations of the Farm Animal Welfare Council (6) and European Food Safety Authority (7) by utilizing animal-based measures where possible. The WQ^®^ assessment is made up of many, diverse animal indicators at both herd and individual level to provide sufficient detail about the welfare status in a herd (8). All indicators included in the WQ^®^ protocol were selected on the basis of scientific evidence for their validity, reliability, and feasibility (9, 10).

The top-down general framework for the WQ^®^ protocol was developed around four welfare principles: A. Good feeding, B. Good housing, C. Good health, and D. Appropriate behavior. Within these four principles are twelve welfare criteria. Due to the absence of a suitable measure, the criterion thermal comfort is not applied to cattle. Within each of the remaining eleven criteria are one to ten welfare measures. An overview is provided in Table 1. Inversely, the integration of WQ^®^ scores follows a bottom-up approach. Based on the relevant measure(s), each criterion is scored on a scale of 0–100, with 100 being the best possible welfare. In turn, these criteria scores are combined to generate a score from 0 to 100 for each of the four principles. These four principle scores are ultimately combined to assign the herd to one of four categories: “Excellent”, “Enhanced”, “Acceptable”, or “Not classified”. A full description of the score integration can be found in the WQ^®^ protocol (1).

**Table 1:** Summary of the structure of Welfare Quality^®^ applied to dairy cattle including principles, criteria, and measures.

**Principle**	**Criterion**	**Measure**
*A. Good feeding*	1. Absence of prolonged hunger	Body condition score
	2. Absence of prolonged thirst	Provision of water
*B. Good housing*	3. Comfort around resting	Behavior at lying
		Cleanliness of cows
	4. Thermal comfort	No measure available
	5. Ease of movement	Possibility for cows to move freely
*C. Good health*	6. Absence of injuries	Lameness
		Integument alterations
	7. Absence of disease	Ocular discharge
		Nasal discharge
		Coughing
		Hampered respiration
		Vaginal discharge
		Diarrhea
		Somatic cell count
		Dystocia
		Downer cows
		Mortality
	8. Absence of pain caused by management procedures	Disbudding/dehorning
		Tail docking
*D. Appropriate behavior*	9. Expression of social behavior	Agonistic behaviors
	10. Expression of other behaviors	Access to pasture
	11. Good human-animal relationship	Avoidance distance test
	12. Positive emotional state	Qualitative behavior assessment

Even if a sufficient sample of representative herds across the country were assessed, a single overall status may mask meaningful regional variations. Such geographical variations in dairy cow welfare have been alluded to in other studies (11, 12). The Norwegian mainland is long, stretching across 13° of latitude. The glaciated terrain consists of rugged mountains broken by fertile valleys. The coastline is relatively mild due to the Gulf Stream while the north is predominantly arctic tundra. As a result, the typical climatic conditions vary between the five regions for which animal welfare data were collected. For example, an area in the west (Vestlandet North) had a 30-year cumulative mean annual precipitation almost two and a half times greater than an area in the southeast (Østlandet South). Similarly, lowland areas in the southeast had a 30 year cumulative mean annual temperature of 6.41°C compared to 0.61°C in mountainous parts of the east (Østlandet North) (13).

We hypothesized that the score for the four overarching WQ^®^ principles would vary between the five regions in which welfare was assessed. Geographical diversity may influence the welfare of cattle in several ways. The amount of time that dairy cows spend outside at pasture each year, the composition of feed available, and the prevailing housing designs (for example, insulated or non-insulated barns) may vary depending on the regional climate. In turn, this variation may influence dairy cow welfare. If welfare is found to vary significantly from region to region, this could have important implications both for the provision of a national status and for targeting regionally specific welfare interventions.

To our knowledge, a primarily animal-based welfare assessment of this scale has never been previously conducted in Norwegian loose-housed dairy herds. Specific topics relevant to dairy cow welfare have been studied in Norwegian dairy herds, for example skin lesions (14); mastitis (15); lameness (16); and body condition (17), but never in combination using an integrated animal welfare assessment protocol. Similarly, regional variations of the welfare status in Norwegian dairy herds have not been formally investigated.

Previous studies have indicated that, contrary to what has happened in other European countries, Norwegian dairy producers have not faced a problem of consumer distrust. Animal welfare in Norway has been primarily governed and regulated by the issuing of statutory regulations that often go beyond EU standards (18). The legislative requirement for pasture access in the 2004 “Regulations for the Keeping of Cattle” (19) is an example of this. The present study will provide insight into whether the reality of animal welfare on-farm, as assessed using the WQ^®^ protocol, aligns with the high legislative standards and consumer expectations in Norway. Such information may be of interest to dairy industry stakeholders in other countries as they consider how to address growing public concern about animal welfare. Additionally, should regional variation be detected, it would facilitate the implementation of regionally specific welfare interventions in Norway. Awareness of the potential for regional variation could prove useful beyond Norwegian conditions, in any country with a welfare status which may similarly vary.

## 2. Materials and methods

### 2.1. Herd selection

The herds were selected from the Norwegian Dairy Herd Recording System (NDHRS), operated by Mimiro AS, a subsidiary of TINE SA. TINE SA is Norway's largest producer, distributor, and exporter of dairy products (20), representing ~97.5% of Norwegian dairy herds. Herds eligible for inclusion in the study were members of TINE SA and participants in NDHRS. The study population was selected to be broadly reflective of Norway's expected future dairy herds by only including loose-housed herds and herds of 25-100 standardized cow-years in size at the time of selection. Herds using tie-stall housing, representing 54.2% of the herds in Norway (21), were excluded as this housing system is banned completely from 2034 onwards (22). In 2021 loose-housing herds accounted for 67.3% of the total number of dairy cows in Norway (21). The average number of standardized cow-years within TINE dairy herds in 2021 was 30.9, increased from 22.1 in 2011 (21), with the desired range of standardized cow-years for inclusion representing the trend toward larger herd sizes in Norway. Geographically, the herds were selected from the municipalities located in nine of the eleven counties in Norway. Herds from the two most northern counties (Nordland and Troms og Finnmark), ~10% of the herds in Norway (21), were excluded due to budgetary constraints.

**Figure 1:**
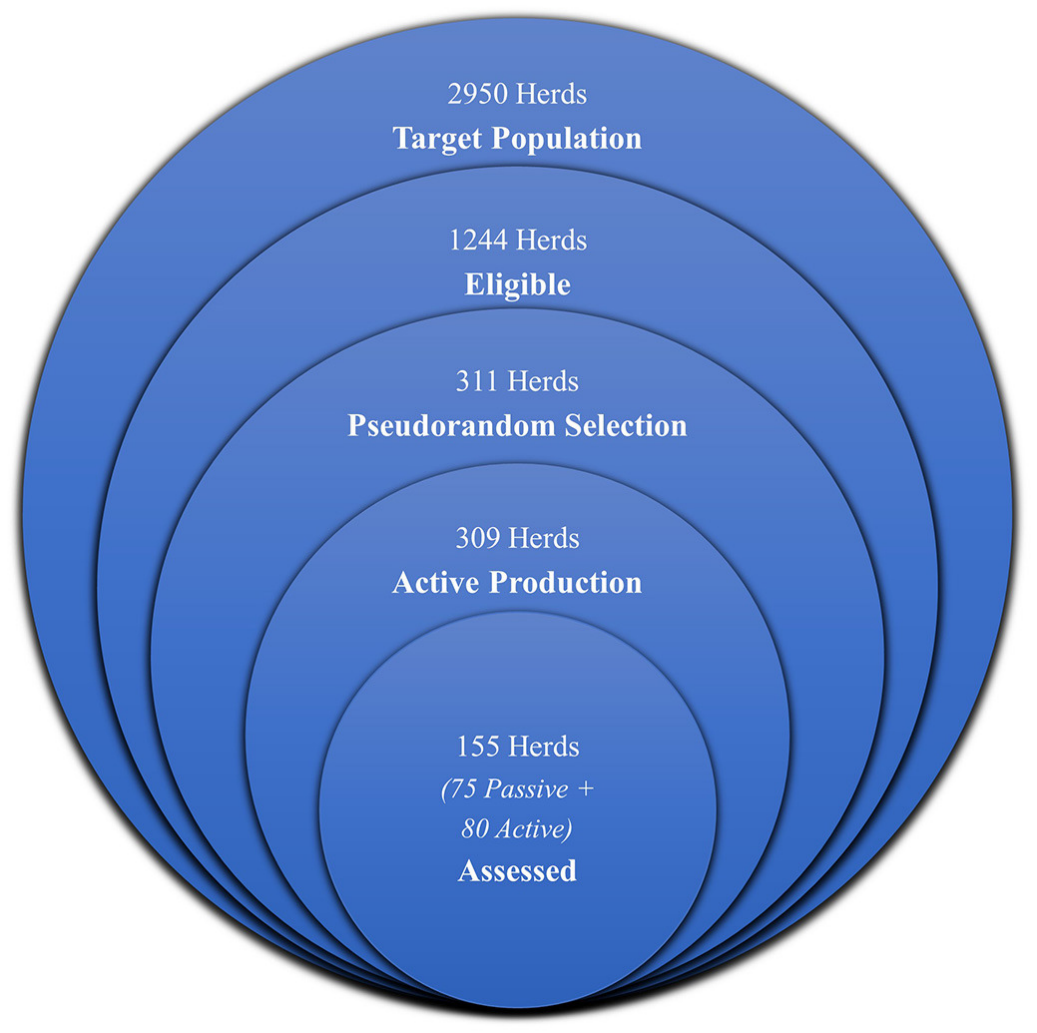
Euler diagram visualizing the selection and recruitment process.

From the target population of all loose-housed Norwegian dairy herds (*n* = 2950) (21) a list was prepared of herds which met the remaining inclusion and exclusion criteria of the project (*n* = 1244) using SAS 9.4 software (23). The list was sorted by producer identification number, the first four digits of which are related to geographical location. A geographically stratified pseudorandom sample of potential herds was chosen from this list by selecting every 4th herd (*n* = 311). Following inspection of the list of selected herds, two herds were deemed ineligible for participation as they were no longer producing milk. A list of active selected herds (*n* = 309) was prepared. Figure 1 summarizes the selection and recruitment process.

To minimize the influence of routine claw trimming on lameness scoring, WQ^®^ specifies that herds should not be assessed until at least 28 days after routine claw trimming. Additionally, for practical reasons, lactating cows must be housed during the visit. Due to these constraints, scheduling practicalities, budgetary limitations, and potential participant hesitancy we anticipated it would be possible to visit ~50% of the eligible selected herds.

### 2.2. Herd recruitment

**Figure 2:**
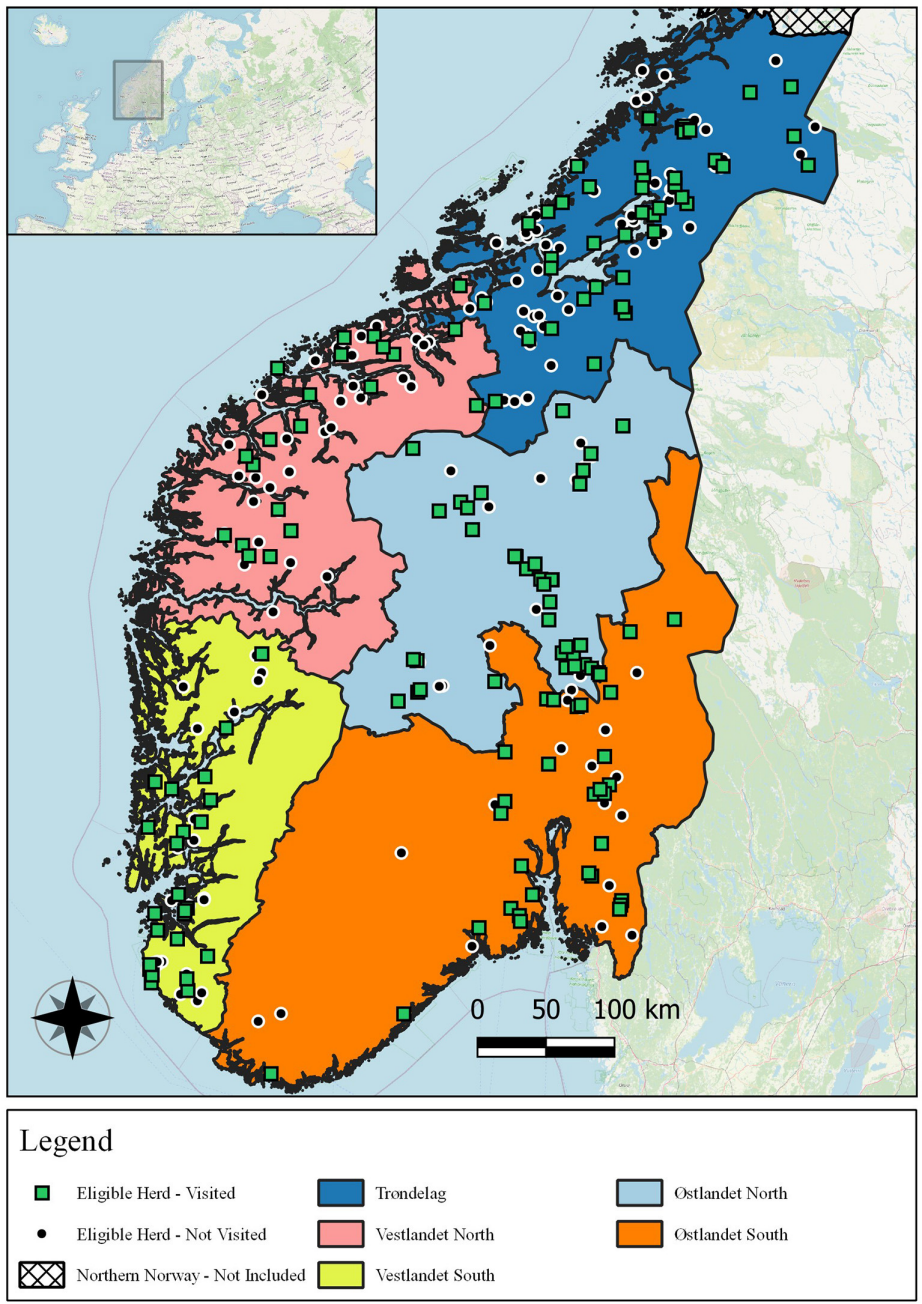
Map of central and southern Norway showing the five regions investigated, the eligible herds which were visited, and the eligible herds which were not visited.

All 155 herds participated voluntarily. Their geographical distribution can be seen in Figure 2. Recruitment occurred over two phases, one passive and one active, as described below. To maximize participation, several methods of contacting producers (e-mail, SMS, telephone call, and word-of-mouth) were attempted.

#### 2.2.1. Passive recruitment

Initially, all 309 active selected herds were emailed by the TINE Member Center, the group responsible for internal communication with members of TINE SA. The email contained information about the project and a link to a web-based questionnaire with four questions (24). After confirming their producer identification number and name, potential participants were asked if they were interested in participating and, if so, they were asked for their preferred contact telephone number. SMS nudges were sent three and eight days after the initial email to remind the potential participants to respond to the questionnaire. We informed the TINE advisors assigned to each of the selected herds about the project by email and asked them to encourage their clients to complete the questionnaire. Herds were classified as passively recruited if they expressed interest in participating through the initial questionnaire.

All active selected herds were plotted geographically using GPS coordinates in Google Maps™ (25). To ensure the sample was broadly representative geographically, the herds were divided into approximate geographical areas. The herds in each area which had expressed interest in participating were contacted by telephone in order of appearance on the list of active selected herds. If possible, a visit was arranged on an appropriate date. When visits were arranged with approximately half of the herds in an area, herds in a new area were contacted.

#### 2.2.2. Active recruitment

In areas where an insufficient number of herds were recruited passively, herds which had not explicitly stated they did not wish to participate in a questionnaire response were contacted by telephone. Their participation was requested and if they were willing a visit was arranged. These herds were classified as actively recruited because their recruitment required a direct telephone conversation rather than expressing interest of their own volition through the questionnaire. They were contacted by the assessors in order of appearance on the list of active selected herds until visits were arranged with ~50% of herds in an area.

### 2.3. Regions

For the purpose of investigating regional variation, the 155 herds were assigned to one of five regions corresponding to their administrative district within TINE SA. These administrative districts are based on traditional geographical divisions of Norway. The five regions were Trøndelag (*n* = 43), Østlandet South (*n* = 38), Østlandet North (*n* = 29), Vestlandet South (*n* = 24), and Vestlandet North (*n* = 21). The five regions and their herds are shown in Figure 2.

### 2.4. Assessor training

Six assessors, three veterinarians and three ethologists were trained to use the WQ^®^ protocol in a three-day course provided by a delegate of the WQ^®^ Network. All assessors had previous experience of handling and body condition scoring dairy cattle. Due to international travel restrictions at the time of training, the theoretical component of the course was delivered online in a classroom environment. Practical aspects of the course were performed locally under remote supervision and the assistance of two assessors who had previously completed additional training. The course concluded with an online assessment of interobserver reliability (IOR) using photographs and videos. The percentage agreement with the silver standard reference values ranged from 73 to 100%. The IOR was evaluated using statistical techniques by the course provider and deemed to be acceptable for all measures except for those related to behavior observations. This was consistent with the prior experience of the course provider. For this reason, extra attention was placed on these behavioral assessments during the shadowed visits which followed the course.

Each assessor performed one to two complete shadowed visits accompanied by a more experienced assessor. These shadow visits occurred prior to undertaking visits independently. Two herds not included in the list of randomly selected herds were used for shadow visits but not included in the analysis. All assessors were subsequently WQ^®^ certified.

### 2.5. Data collection

A full WQ^®^ assessment was performed for each herd between April 2021 and January 2022 as described by the WQ^®^ protocol (1). All of the herds were managed in a broadly similar manner. Cows were housed indoors on rubber-matted cubicles for most of the year with variable amounts of pasture access during the summer months. Cows were milked using either automatic milking systems (AMS) or milking parlors. Forage was provided *ad libitum* at a feed face with supplies being refreshed up to several times daily. Concentrate feeding took place primarily in the AMS or milking parlor, with additional concentrate feed often being provided by concentrate feed dispensers.

The welfare assessment data were recorded either on paper using the protocol's example recording sheets; using an Excel recording sheet; or using an online application developed for the purposes of data collection and WQ^®^ score calculation (26). Sick or freshly calved cows were excluded. The data collected for each measure and the sources of the data are summarized in Table 2. For measures based on individual animals the sample size depended on the size of the herd. The number of individuals recommended by the WQ^®^ protocol (1) were chosen using systematic sampling by selecting every second animal at the feed face (28) while performing the avoidance distance test. Simultaneously, each animal was alternately designated to clinical assessment of its left or right side. Sampling was divided between at least two separate time periods. The same individuals were assessed for avoidance distance and clinical measures whenever possible.

**Table 2:** Summary of the data collected for each measure of the Welfare Quality^®^ protocol and the sources of the data (^*^on-farm observation or clinical assessment; ^**^interview with farmer or livestock manager; ^***^Norwegian Dairy Herd Recording System).

**Measure**	**Associated data and source**
Body condition score	% very lean cows^*^[corresponding to a score < 2.5 in the grid of Edmonson et al. (27)]
Provision of water	Number of drinkers^*^; length of water troughs^*^; cleanliness of water points^*^; water flow rate^*^
Behavior at lying	Mean time to lie down^*^; % cows colliding with housing equipment when lying down^*^; % of cows lying partly or completely outside of the lying area^*^
Cleanliness of cows	% cows with dirty udder^*^; % cows with dirty hindquarters^*^; % cows with dirty lower legs^*^
Thermal comfort	Criterion not assessed
Possibility for cows to move freely	Presence of tethering^*^
Lameness	% mildly lame cows^*^; % moderately or severely lame cows^*^
Integument alterations	% cows with mild integument alterations (hairless patch)^*^; % cows with severe integument alterations (lesion or swelling)^*^
Ocular discharge	% cows with ocular discharge^*^
Nasal discharge	% cows with nasal discharge^*^
Coughing	Number of coughs per cow per 15 min^*^
Hampered respiration	% cows with hampered respiration^*^
Vaginal discharge	% cows with vaginal discharge^*^
Diarrhea	% cows with diarrhea^*^
Somatic cell count	% cows with somatic cell count >400,000 in the previous three milk recordings^***^
Dystocia	% cows affected by dystocia in the previous 12 months^**^
Downer cows	% cows recumbent for more than 24 h in the previous 12 months^**^
Mortality	% cows dead, euthanized, or emergency slaughtered on-farm in the last 12 months^**/***^
Disbudding/dehorning	Procedure used for disbudding/dehorning^**^; use of anesthetics^**^; use of analgesics^**^
Tail docking	N/A in Norway as forbidden as a routine management procedure
Agonistic behaviors	Number of headbutts per cow per hour^*^; number of displacements, bouts of fighting, and occurrences of chasing or chasing up per cow per hour^*^
Access to pasture	Number of days per year with access to pasture^**^; number of hours per day with access to pasture^**^
Avoidance distance test	% cows which can be touched^*^; % of cows which can be approached between 10 and 50 cm^*^; % cows that can be approached between 60 and 100 cm^*^; % of cows that cannot be approached closer than 110 cm^*^
Qualitative behavior assessment	Values for 20 descriptors (0–125mm scale)^*^

The vast majority of cows assessed were Norwegian Red (NRF). The body condition scoring system used by WQ^®^ is dependent on whether the cow is of dairy or dual-purpose type. To account for neither of the WQ^®^ protocol's body condition scoring systems being fully suitable for NRF, a previously published five-point body condition scoring grid (27) adjusted for NRF (29) was used to assess body condition in increments of half points. The body condition score for NRF was recorded as a WQ^®^ body condition score registration using cut-off points agreed with a member of the WQ^®^ Network. “Very lean” corresponded to a score < 2.5, “Normal” was 2.5–3.5, and “Very fat” was >3.5. The body condition of other breeds was scored according to the WQ^®^ system.

Some data were self-reported by the farmer from on-farm records or memory. These included the number of cases of dystocia, the number of downer cows, and the number of cows which died, were euthanized or were emergency slaughtered on-farm during the 12 months prior to the visit. Additionally, the number of days of pasture access per year and the number of hours of pasture access during those days was self-reported. The definition of pasture extended to any vegetation covered outdoor area, even if it did not contribute to the cows' nutritive requirements. Self-reporting was preferred as this aligned most accurately with the WQ^®^ definitions.

When dystocia data were missing in the dataset (*n* = 7), the mean value from the other herds was used instead as this was considered by the authors to be broadly reflective of Norwegian conditions. When mortality data were unavailable through self-reporting, the mortality records for the farm were accessed through the NDHRS and these data used. For every herd, data for the average number of lactating animals and the number of calvings which took place in the herd during the 12 months prior to the visit were retrieved from the NDHRS following the on-farm assessment. These data were used to calculate the percentage mortality, dystocia, and downer cows during that time period. Somatic cell count (SCC) data for each cow for the last three milk recordings prior to the visit were also obtained from NDHRS and the percentage of cows with a SCC ≥400,000 cells per milliliter in at least one of these milk recordings was calculated.

### 2.6. Welfare Quality^®^ score calculation

The four principle and eleven applicable criteria scores were calculated based on the formulae provided in the updated version of the WQ^®^ protocol (1). These formulae were programmed into an online application designed for the purpose of data collection and calculation of integrated WQ^®^ scores (26). All data collected using paper recording sheets or the Excel sheet were transferred into the online application's database for the purpose of score calculation. All of the raw data and the calculated scores were exported from the database in.csv format for analysis. The application automatically assigned each herd to a WQ^®^ category based on which aspiration thresholds defined by the WQ^®^ protocol were met by its principle scores. Herds were classified as “Excellent” if all principle scores were over 55 and two were over 80; “Enhanced” if all principles scores were over 20 and two were over 55; “Acceptable” if all principles scores were over 10 and three were over 20; and “Not classified” if they did not meet the requirements for “Acceptable”.

### 2.7. Statistical analysis

All data analyses were performed using Stata 16 (30). The exact operations used in Stata stand in quotation marks and are italicized. All 155 herds visited were included in the analyses.

#### 2.7.1. Descriptive statistics

The integrated WQ^®^ scores (4 principles and 11 criteria) and observed prevalences of common welfare issues and other welfare related herd level summary data (*n* = 34) were reported in table format, organized within the structure of WQ^®^ as described in Table 1. To provide more detailed descriptions of the data, data distributions and the relationships between variables were assessed graphically as necessary. Examples include the relationship between ocular and nasal discharge and the month of the visit, as well as the linear relationship between the proportion of very lean animals and the proportion of dairy-type animals on-farm.

#### 2.7.2. Statistical modeling and regional variation

Generalized linear modeling (GLM) (31) was used to investigate whether the welfare status varied depending on region. In total, four models were built, one for each principle: A. Good feeding, B. Good housing, C. Good health, and D. Appropriate behavior. The dependent variable for each model was the eponymous continuous principle score ranging from 0 to 100. As region was the variable of primary interest, a categorical independent variable denoting the five regions was included in all four models as a fixed effect.

The distribution of the dependent variable and its transformations, based on a subset of the ladder of powers (32), were screened initially using the “*gladder*” command. The link function corresponding to the most normally distributed transformation was included as a component of the model. The Gaussian family function was included in all four models due to the distribution of the dependent variable or its chosen transformation.

Each model was built using a forward selection process. The relationship between the independent variables and the dependent variables was assessed univariably. A conservative initial inclusion criterion of *P* < 0.20 was used during univariable screening, however, biological plausibility took precedence throughout the model building process. Independent variables were retained following univariable screening process regardless of their statistical relationship to the dependent variable if it was biologically justifiable. Potential biological relationships were identified using directed acyclic graphs (33).

The independent variables assessed univariably were assessor (coded 1–6); milking system (AMS or parlor); recruitment method (passive or active), and herd size (continuous). Herd size, as the only continuous independent variable, was plotted against the dependent variable using locally weight scatterplot smoothing (LOWESS) (34) to assess whether a linear relationship was present or not prior to univariable screening. Such a relationship only existed between herd size and the principle B. Good housing.

Despite training and IOR testing, significant assessor effects were detected during univariable screening for the models B. Good housing, C. Good health, and D. Appropriate behavior. Furthermore, the distribution of herds visited by different assessors was not even across the five regions. It was concluded that assessor effects could act as a confounder for potential regional effects. Therefore, it was necessary to include the assessor variable in all models intended to determine the extent of regional variation.

During multivariable modeling, independent variables carried forward from the univariable screening were retained if they improved the explanatory power of the model and were biological plausible, resulting in a best fitted model for each principle. The best fitted models are reported in Table 3. As all models were nested during the building process, the models were assessed using Wald Chi^2^ Test scores and their significance values (35) to determine the best fitted model.

**Table 3:** Details of the four best fitted generalized linear models (A. Good feeding, B. Good housing, C. Good health, and D. Appropriate behavior) used to investigate regional variation.

**Welfare Quality^®^ Principle (Dependent Variable)**	**Best Fitted Model (Independent Variables)**	**Model Intercept (Scale 0–100)**	**Std. Err. (Intercept)**	**Homoscedasticity**	**Normality**	**Link**	**Family**	**Wald Chi^2^ Test (Prob > Chi^2^)**
*A. Good feeding*	i.region i.assessor	60.5	4.3	Yes	Yes	Identity	Gaussian	11.0 (0.3)
*B. Good housing*	i.region i.assessor herd_size	58.8	2.3	Yes	Yes	Identity	Gaussian	52.2 (< 0.01)
*C. Good health*	i.region i.assessor i.recruit i.milking_system	37.8	1.0	Yes	Yes	Log	Gaussian	46.52 (< 0.01)
*D. Appropriate behavior*	i.region i.assess i.recruit	49.5	1.7	Yes	Yes	Identity	Gaussian	16.6 (0.1)

Post estimation, the linear prediction and deviance residuals of each model were calculated. Homoscedasticity was assessed by plotting these two sets of residuals against each other on a two-way scatterplot. Normality of the deviance residuals was assessed visually by two methods. First by producing a histogram and secondly by plotting the quantiles of the deviance residuals against the quantiles of normal distribution. The details of the intercepts of the best fitted models used, the standard errors of their intercepts, their post estimation results, and their Wald Chi^2^ Test scores with significance values are provided in Table 3.

The “*margins*” command with region as the factor variable was used to calculate the estimated mean for each of the five regions based on the best fitted models A. Good feeding, B. Good housing, C. Good health, and D. Appropriate behavior. The estimated means were summarized on a regional basis in table format. Where a significant difference was found between regions, this was demarcated in the table in bold. The significance threshold was set at *P* = 0.05. Additionally, they were visualized with their 95% confidence intervals using the “*marginsplot*” command.

## 3. Results

### 3.1. Participating herds

The 155 herds assessed had an average herd size of 52 cows with a range from 18 to 117 cows. AMS were present in 89% of the herds (*n* = 138) and the remaining 11% used milking parlors (*n* = 17). The proportion of the cow assessed for individual level measures was between 46 and 100% per herd. On average, 74% of the eligible lactating cows and heifers in each herd were assessed individually. The mean proportion of NRF in the herds assessed was 92.7%. There were slightly more actively recruited herds (51.5%) than passively recruited herds (48.4%).

### 3.2. Welfare Quality^®^ categories

More than half of the herds, 55.5% (*n* = 86), were classified as “Enhanced” based on their WQ^®^ assessment. Of the remaining herds, 43.2% (*n* = 67) were placed in the “Acceptable” category and 1.3% (*n* = 2) were deemed “Unclassified”. None of the herds attained the highest WQ^®^ classification of “Excellent”.

### 3.3. Welfare Quality^®^ scores and measures

Welfare Quality^®^ principle and criteria scores are shown in Table 4. The observed prevalences of common welfare problems as well as other herd level welfare measures not expressed as prevalences are displayed in Table 5.

**Table 4:** Summary of Welfare Quality^®^ scores in Norwegian loose-housed dairy herds (*n* = 155) showing the mean, standard deviation of the mean, median, maximum, and minimum for each value.

**Welfare Quality^®^ Principles & Criteria**	**Mean**	**Std. dev**.	**Median**	**Min**.	**Max**.
*A. Good feeding*	56.0	27.8	60.9	9.1	100.0
1. Absence of prolonged hunger	83.5	19.7	85.7	19.3	100.0
2. Absence of prolonged thirst	54.9	32.7	60.0	3.0	100.0
*B. Good housing*	63.4	9.4	63.8	40.2	93.3
3. Comfort around resting	41.9	14.9	42.5	5.0	89.3
4. Thermal comfort	–	–	–	–	–
5. Ease of movement	100.0	0.0	100.0	100.0	100.0
*C. Good health*	38.1	8.2	37.2	20.0	66.2
6. Absence of injuries	43.0	13.5	42.9	14.6	88.9
7. Absence of disease	35.0	14.3	33.3	10.0	86.0
8. Absence of pain induced by management procedures	75.0	0.0	75.0	75.0	75.0
*D. Appropriate behavior*	47.6	10.0	48.3	16.3	67.5
9. Expression of social behaviors	53.9	18.9	54.4	9.3	96.8
10. Expression of other behaviors	47.7	16.3	53.6	0.0	100.0
11. Good human-animal relationship	77.7	12.6	79.2	35.6	97.4
12. Positive emotional state	49.9	12.7	51.5	15.0	79.6

Scale 0–100.

**Table 5:** Summary of observed prevalences and other herd-level registrations related to animal welfare in Norwegian loose-housed dairy herds (*n* = 155) showing the mean, standard deviation of the mean, median, maximum, and minimum for each value.

**Welfare Value**	**Mean**	**Std. dev**.	**Median**	**Min**.	**Max**.
Very lean body condition score	3.0 %	4.9 %	1.9 %	0.0 %	34.5 %
Very fat body condition score	12.1 %	12.1 %	9.1 %	0.0 %	61.3 %
Mean time to lie down in seconds	6.2	0.9	6.1	4.3	9.4
Collisions with housing equipment when lying down	11.4 %	14.2 %	7.7 %	0.0 %	90.0 %
Lying partly or completely outside the lying area	2.8 %	4.6 %	0.9 %	0.0 %	25.5 %
Dirty udder	17.0 %	14.0 %	13.3 %	0.0 %	76.6 %
Dirty hindquarter	33.7 %	21.5 %	31.8 %	0.0 %	94.6 %
Dirty lower hind legs	62.6 %	23.6 %	64.0 %	4.9 %	100.0 %
Mildly lame cows	11.1 %	7.5 %	9.4 %	0.0 %	36.4 %
Moderately or severely lame cows	2.9 %	4.0 %	2.3 %	0.0 %	25.0 %
Mild integument alterations	45.3 %	13.8 %	46.3%	4.3 %	78.6 %
Severe integument alterations	32.6 %	16.9 %	30.0%	0.0 %	86.4 %
Tarsal hairless patches	47.7 %	15.9 %	48.5 %	8.9 %	79.5 %
Tarsal lesions	7.4 %	7.8 %	4.3 %	0.0 %	41.0 %
Tarsal swellings	4.2 %	7.9 %	2.5 %	0.0 %	66.7 %
Carpal hairless patches	29.4 %	13.7 %	27.3 %	0.0 %	71.9 %
Carpal lesions	1.5 %	3.5 %	0.0 %	0.0 %	20.0 %
Carpal swellings	4.7 %	5.2 %	3.1 %	0.0 %	27.3 %
Neck/shoulder/back hairless patches	8.0 %	8.4 %	5.4 %	0.0 %	33.3 %
Neck/shoulder/back lesions	0.5 %	1.5 %	0.0 %	0.0 %	10.3 %
Neck/shoulder/back swellings	5.1 %	10.0 %	0.0 %	0.0 %	65.9 %
Ocular discharge	15.8 %	13.8 %	13.4 %	0.0 %	60.0 %
Nasal discharge	6.8 %	7.6 %	3.6 %	0.0 %	41.7 %
Vaginal discharge	0.9 %	1.7 %	0.0 %	0.0 %	11.1 %
Diarrhea	8.8 %	10.6 %	6.1 %	0.0 %	57.1 %
Hampered respiration	0.3 %	0.01 %	0.0 %	0.0 %	12.9 %
Dystocia	3.3 %	2.9 %	2.6 %	0.0 %	18.5 %
Downer cows	1.3 %	1.8 %	0.0 %	0.0 %	8.0 %
Mortality	4.5 %	3.6 %	3.6 %	0.0 %	16.7 %
Somatic cell count >400,000 in the previous three milk recordings	15.2 %	6.9 %	14.0 %	0.0 %	33.3 %
Number of coughs/cow/15 min	0.10	0.09	0.08	0.00	0.42
Number of headbutts/cow/hour	0.74	0.42	0.66	0.00	2.23
Number of displacements (including fighting, chasing, and chasing up)/cow/hour	0.72	0.41	0.61	0.00	2.19
Number of days/year with 8 or more hours of access to pasture	109	47	120	0	365
0 cm avoidance distance test score	59.8 %	19.4 %	61.5 %	6.7 %	94.1 %
10–50 cm avoidance distance test score	33.7 %	14.9 %	32.5 %	5.9 %	71.7 %
60–100 cm avoidance distance test score	5.6 %	7.1 %	3.2 %	0.0 %	43.3 %
110–200 cm avoidance distance test score	0.9 %	2.0 %	0.0 %	0.0 %	10.0 %

#### 3.3.1. Good feeding

The mean score for the principle good feeding across all herds was ~56 but this varied hugely from < 10 to the maximum of 100 between herds. As can be seen in Table 4, the standard deviation of the mean herd score for the principle good feeding was far higher than the standard deviation of the mean for the other three principle scores. Good feeding was, therefore, the principle with the greatest variation between herds.

##### 3.3.1.1. Absence of prolonged hunger and thirst

The criterion absence of prolonged hunger was scored, on average, higher than the mean score for the criterion absence of prolonged thirst. Its minimum was over 16 points higher. The scores for absence of prolonged hunger were skewed to the left, indicating that the majority of herds did not have many cows experiencing prolonged hunger. Despite this, the scores for this criterion were variable, with the lowest scoring herd achieving less than 20 points. The criterion absence of prolonged thirst had the highest standard deviation of the mean of any of the WQ^®^ scores which contributed to the herd variation seen at the principle level for good feeding.

###### 3.3.1.1.1. Body condition score

The criterion for absence of prolonged hunger is calculated based on the proportion of cows in each herd with a very lean body condition score. As shown in Table 5, the mean herd prevalence for ‘very lean' cows was just under 3%. This data was heavily right skewed, with only 21% of herds having more than 5% ‘very lean' animals. Even so, in one herd more than one third of the animals were classified as ‘very lean', a clear example of variation between herds. On the other hand, the mean herd prevalence for ‘very fat' cows was just over 12%. Therefore, the mean herd prevalence of cows with a normal body condition score was 85%. At herd level, LOWESS showed a positive linear relationship between the proportion of very lean animals and the proportion of cows classified as dairy-type breeds by WQ^®^.

#### 3.3.2. Good housing

The mean score for the principle good housing was just above 63 points, as shown in Table 4. No herds scored below 40 points for this principle, but this is because each herd scored maximum points for the contributing criterion ease of movement. Less variation from herd to herd, at principle level, was seen as a result. The range of scores for this principle was smaller (53 points) than the range for the principle good feeding. Furthermore, the standard deviation of the mean for this principle was one third of the size of the standard deviation of the mean for the principle good feeding.

##### 3.3.2.1. Comfort around resting

Comfort around resting was the only criterion that could vary within the good housing principle. The criterion thermal comfort lacked a suitable measure, so it was excluded from the assessment. The exclusion of tie-stall herds from the study resulted in the full score awarded to every herd for the criterion ease of movement. The consistent high scoring of the ease of movement criterion appears to have compensated, at a principle level, for the relatively low scoring for the criterion comfort around resting. Nearly three quarters of the herds scored < 50 points for comfort around resting and two herds scored < 6 points.

###### 3.3.2.1.1. Time needed to lie down

WQ^®^ assigns the mean time needed to lie down for each herd to one of three categories: normal (< 5.2 s); moderate problem (5.2–6.3 s); or severe problem (>6.3 s). Of the herds assessed, 15.5% of herds were classified as normal, 41.3% as having a moderate problem, and 43.2% a severe problem. That herds were classified across all three categories highlights the variation at herd level. The mean time across all herds was only slightly below the border between a moderate and a severe problem.

###### 3.3.2.1.2. Animals colliding with housing equipment during lying down

As can be seen in Table 5, there was a large amount of variation between herds for this measure. The data was skewed heavily toward 0%, suggesting this was not an issue for the majority of herds, but a number of herds did have problems with cows colliding with housing equipment during lying down movements. In 15 of the herds, over 30% of lying down movements resulted in collisions. These herds are classified by WQ^®^ as having a severe problem. In one herd in particular, nine out of ten recorded lying down movements resulted in a collision.

###### 3.3.2.1.3. Animals lying partially or completely outside of the lying area

The proportion of animals in each herd observed lying partly or completely outside the lying area was similarly skewed toward lower prevalences. A little over one fifth of the herds assessed were classified by WQ^®^ as having a severe problem, meaning they had more than 5% of their animals lying partly or completely outside the designated lying area. While a quarter of cows in one herd were recorded as lying out, more than half of the herds had < 3% of cows lying partially or completely outside the lying area as demonstrated by the median score in Table 5.

###### 3.3.2.1.4. Cleanliness

The scoring for all three cleanliness measures followed an approximately normal distribution. As described in Table 5, lower hind legs were most commonly scored as dirty, followed by upper hind legs and udders, respectively. Based on the WQ^®^ cleanliness classifications, the mean prevalence for all herds for dirty lower and upper hindlegs would be classified as a severe problem. The mean prevalence of dirty udders would be classed as a moderate problem. There was not a single herd where all of the lower hind legs were scored as clean, with all herds having some cows with dirty lower hind legs. In 21 herds over 90% of the cows were scored as having dirty lower hind legs.

#### 3.3.3. Good health

Table 4 showed that this was consistently the lowest scored principle across the herds and the only principle to have a mean score below 40 points. It also showed the least variation, with the lowest standard deviation of the mean of the four principle scores. No herd achieved a score higher than 67 points.

##### 3.3.3.1. Absence of injuries

Injuries were frequently observed, as can be seen in Table 5. On average, 14.0% of cows in each herd were scored as being lame to some extent, either mildly or moderately/severely. Integument alterations were even more frequent, with 78.2% of individual animals assessed having at least one hairless patch, lesion or swelling on their body. More than half of the affected animals were scored as having mild changes to the integument, meaning they had at least one hairless patch but no lesions or swellings. As can be seen in Table 5, changes in the tarsal region were by far the most common, followed by carpal changes and changes to the neck, shoulder, and/or back. No herds were found to be completely free of integument alterations. The herd prevalences for these measures were variable between herds, especially for tarsal hairless patches, carpal hairless patches, and swellings on the neck, shoulder, and/or back which all had ranges >65%. Hairless patches and swellings on the neck, shoulder, or back, for example, were absent in the majority of herds but very frequent in certain herds.

##### 3.3.3.2. Absence of disease

The criterion absence of disease is made up of ten disease-related measures which are reported in Table 5. These measures are compared to threshold values defined by WQ^®^, “warning” and “alarm”. If the warning threshold is surpassed, it indicates that there may be a problem in the herd, and it could be beneficial to investigate this further. If the alarm threshold is breached, then it is recommended by the WQ^®^ protocol that action be taken to address the problem identified.

High somatic cell count and ocular discharge, both clinical signs of disease issues, were most commonly identified. The mean herd prevalences of nasal discharge, diarrhea, dystocia, downer cows, and mortality were all < 10%. Vaginal discharge and hampered respiration were very rarely encountered. On average, 0.1 coughs were detected per cow per 15 minutes of observation time. The prevalence of these clinical measures varied between herds. For all contributary measures, there were herds in which no animals scored were affected by that particular issue. In other herds, as many as half of all animals scored were affected, depending on the measure.

As shown in Table 5, the mean values for ocular discharge and diarrhea were both higher than their alarm thresholds, 6 and 6.5%, respectively. The mean values for nasal discharge, high somatic cell count, dystocia, and mortality did not meet their alarm thresholds but did surpass their warning thresholds of 5, 8.75, 2.75, and 2.75%, respectively. Other values which contributed to the criterion for absence of disease (downer cows, vaginal discharge, and hampered respiration) were below their warning thresholds.

###### 3.3.3.2.1. Somatic cell count

The mean prevalence of cows with a somatic cell count >400,000 in at least one of last three recordings prior to the visit was over 15%. This exceeded the warning threshold, as described above, and was just below the alarm threshold of 17.5%.

###### 3.3.3.2.2. Ocular and nasal discharge

The prevalences of nasal and ocular discharge showed a seasonal pattern when plotted against the month of the visit. Both increased during the late spring and summer months before decreasing in the autumn.

##### 3.3.3.3. Absence of pain induced by management procedures

The criterion score for absence of pain induced by management procedures was the same for all 155 herds assessed.

#### 3.3.4. Appropriate behavior

The scoring of this principle was approximately normally distributed and varied between herds through a range of over 50 points. Of the four criteria contributing to this principle, good human-animal relationship was the most highly scored, followed, respectively, by expression of social behaviors, positive emotional state, and expression of other behaviors.

##### 3.3.4.1. Expression of social behaviors

The criterion expression of social behavior is scored based on the number of agonistic behaviors observed per cow per hour. These behaviors are divided into two measures, headbutts and displacements, with fighting, chasing, and chasing-up being considered together with displacements. The scores obtained varied from herd to herd through almost the entire possible range of scores. The data were approximately normally distributed.

##### 3.3.4.2. Expression of other behaviors

On average, the herds assessed allowed cows out to pasture for approximately three and a half months per year. The number of days varied from herd to herd, with two herds allowing their cows year-round access to a vegetation-covered outdoor area and five herds (at least one in each region) providing nothing that would be described by WQ^®^ as pasture. This was the only WQ^®^ score in the study that spanned the full possible range from 0 to 100 points.

##### 3.3.4.3. Good human-animal relationship

The criterion score for good human-animal relationship is based on the avoidance distance testing of a sample of individual cows. The outcome of each individual avoidance distance test is assigned by WQ^®^ to one of four categories: the cow could be touched; the cow could be approached close than 50 cm but not touched; the cow could be approached between 50 and 100 cm; or the cow could not be approached closer than 110 cm. On average, almost two thirds of cows could be touched by the assessor. Approximately one third of individual cows could be approached closer than 50 cm but not touched. Less than 1% of cows could not be approached closer than 100 cm. The human-animal relationship did vary from herd to herd, however, as despite nearly half of herds scoring over 80 points, eight herds scored < 50 points.

##### 3.3.4.4. Positive emotional state

The criterion score for positive emotional state was based on qualitative behavioral analysis of the herd. The scoring was approximately normally distributed and varied between herds with a range of 64.6 points from minimum to maximum.

### 3.4. Regional variation

**Figure 3:**
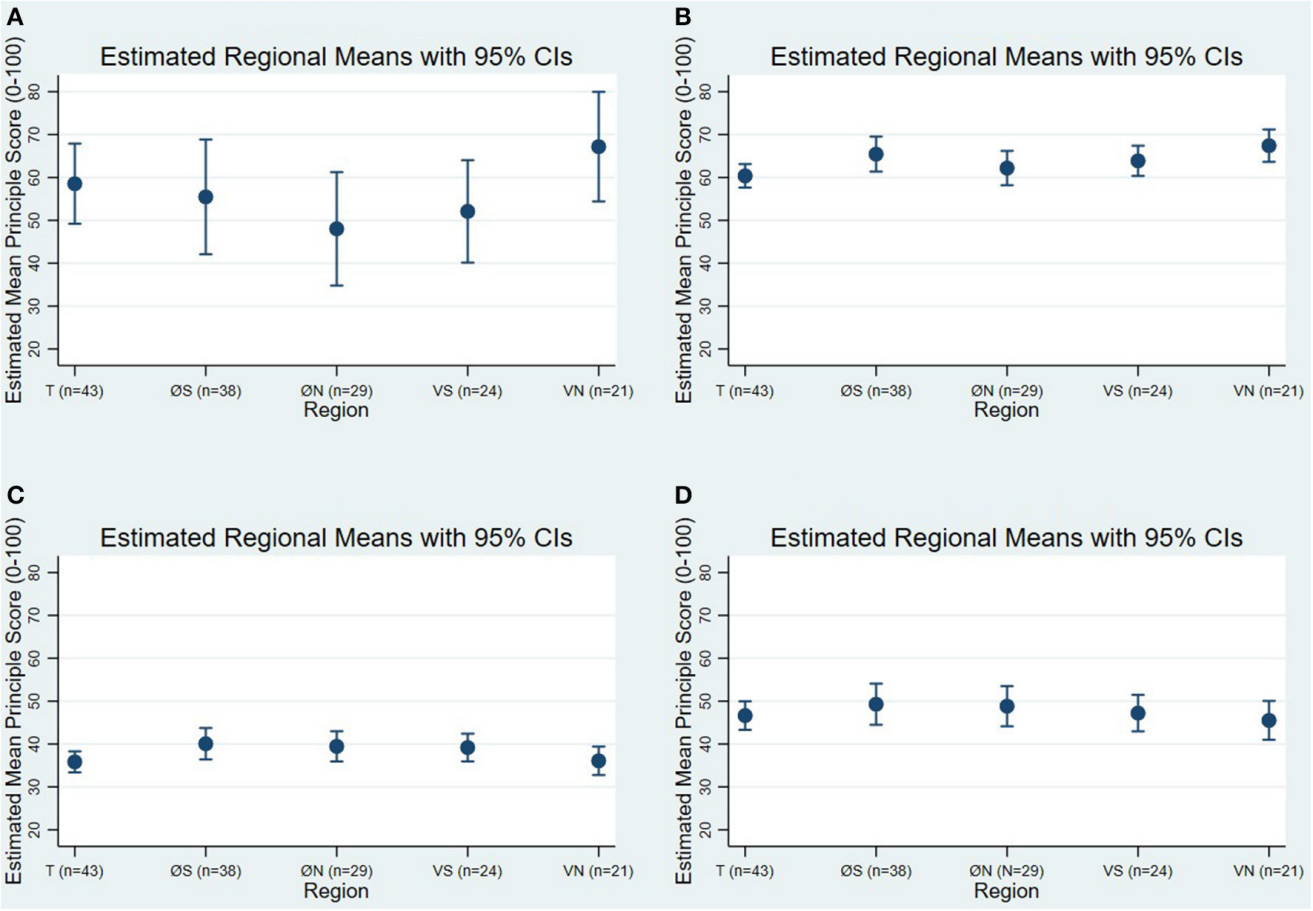
Margins plots visualizing the estimated mean scores for the five regions and their 95% confidence intervals (CIs) based on the best fitted generalized linear models **(A)** Good feeding, **(B)** Good housing, **(C)** Good health, and **(D)** Appropriate housing, as described in Table 3. The regions are labeled as follows: T, Trøndelag, 43 herds; ØS, Østlandet South, 38 herds; ØN, Østlandet North, 29 herds; VS, Vestlandet South, 24 herds; VN, Vestlandet North, 21 herds.

The estimated mean WQ^®^ principle scores for the five regions are reported in Table 6. The only principle that showed significant regional variation in the estimated mean principle scores was B. Good housing. The estimated mean score for Trøndelag was over 7 points lower than the mean score which was estimated for Vestlandet North. These estimated mean scores are demarcated in Table 6 in bold. Figure 3 visualizes the mean scores for region with their 95% confidences intervals. Visual assessment of the 95% confidence intervals for these two regions for the principle B. Good housing demonstrates the significant difference. The estimated mean scores for the principles A. Good feeding, C. Good health, and D. Appropriate behavior did not vary significantly between the five regions.

**Table 6:** Summary of estimated mean regional Welfare Quality^®^ principle scores based on the generalized linear models A. Good feeding, B. Good housing, C. Good health, and D. Appropriate behavior.

**Welfare Quality^®^ Principle**	**Trøndelag (*n* = 43)**	**Østlandet S (*n* = 38)**	**Østlandet N (*n* = 29)**	**Vestlandet S (*n* = 24)**	**Vestlandet N (*n* = 21)**
*A. Good feeding*	58.5	55.5	48.0	52.1	67.2
*B. Good housing*	**60.3**	65.5	62.1	63.9	**67.5**
*C. Good health*	35.9	40.1	39.5	39.2	36.1
*D. Appropriate behavior*	46.6	49.3	48.8	47.2	45.5

Scale 0–100. Estimated mean scores which vary significantly on a regional level are demarcated in bold.

## 4. Discussion

### 4.1. Overall status

Our objective was to report the status of animal welfare in Norwegian loose-housed dairy herds based on assessments made using the WQ^®^ protocol. Just over half of the herds were classified as “Enhanced”, the second highest ranking within the WQ^®^ assessment framework. The absence of any herds classified as “Excellent” is similar to the findings of other European studies (11, 36–38). Only two herds were categorized as “Unclassified”, the lowest ranking. These herds had relatively low scores for the criterion absence of prolonged hunger and very low scores for the criterion absence of prolonged thirst compared to the other herds assessed. These features, higher proportions of very lean cows and water access defined by WQ^®^ as insufficient, were consistent with herds classified as “Unclassified” in a study in the Netherlands (36). That study demonstrated that improving water provision was the most effective way for lower ranking herds to move into a higher category while improvements in other aspects did little to improve the overall classification. The authors of that study highlighted that the WQ^®^ categories are overly influenced by certain measures, in particular water provision, and poorly sensitive for other measures, such as those contributing to good health. They suggested that this over sensitivity in one aspect, combined with a lack of sensitivity in other aspects, may lead to increased focus on areas that improve the category of the herd at the expense of diverting attentions away from other important welfare issues (36). Even so, herds categorized as “Unclassified” are likely to be experiencing several important welfare issues in addition to insufficient water provision.

It is important to acknowledge that the herds assessed may not be wholly representative of the target population of Norwegian loose-housed dairy herds due to the exclusion of herds in the two most northern counties, herds that are not members of TINE and the NDHRS, and herds that were outside of the criteria for herd size.

Participation bias, an acknowledged limitation in research where participants take part of their own free will (39, 40), is another limitation of this study. Our findings are potentially biased toward those with an active awareness of, and an interest in, animal welfare. Simultaneously, those who wish to hide what they perceive to be poor animal welfare can do so. Unfortunately, we were limited in our ability to avoid participation bias in our study as only willing participants could be included.

Throughout this discussion, comparisons will be made between the results of this study and those from previous Norwegian studies or studies from other countries. The authors acknowledge that these comparisons must be interpreted cautiously due to methodological differences. We are, however, of the opinion that the context they provide is still of value, particularly where the application of the WQ^®^ protocol has been consistent.

### 4.2. Encouraging findings

On the criteria level, there were several examples of potential welfare issues being well controlled. These included the absence of prolonged hunger, absence of pain due to management procedures, and good human-animal relationship. These three criteria were particularly highly scored in Norway.

The prevalence of very lean animals was higher in this study than has previously been reported in Norway (17). The previous findings were recorded at first insemination which may cause the cows most at risk of low body condition to be under-represented due to acyclicity or management decisions (41). The average proportion of cows classified as very lean was lower in this study than has been seen in studies from other countries (11, 36, 38). From a welfare perspective, a body condition score classified as very lean by WQ^®^ is universally unacceptable for any cow at any stage of lactation (10).

The cows assessed using the avoidance distance test were, by and large, trustful of humans. Similar results have been seen in Austrian dairy herds (42). The assessors were able to approach the vast majority of cows closer than 50 cm before they withdrew, reflective of a good human-animal relationship in a majority of the dairy herds. Cows' relationship to humans is defined as their perception of humans based on their previous interactions. In addition to genetics, a good human-animal relationship is the result of appropriate behavior by stockpeople and infrequent unpleasant interactions between humans and animals. The presence of positive experiences and absence of negative experiences allows cows to develop trust and confidence in humans (43). A positive relationship between dairy cows and humans has been linked to increased cow productivity and safety in the farm working environment (43–45).

### 4.3. Welfare challenges

Based on the WQ^®^ criteria scores, the biggest welfare challenges in Norwegian dairy herds were clinical signs of disease, injuries, compromised resting comfort, and limited time at pasture. Absence of disease scored the lowest on average of all criteria and could be seen as the biggest welfare challenge from a WQ^®^ perspective. This is surprising as the health status of Norwegian cattle is generally considered to be high. Norway is free from several endemic infectious diseases of cattle such as tuberculosis, paratuberculosis, bovine viral diarrheoa, and infectious bovine rhinotracheitis. A concerted effort by the Norwegian dairy industry over the last 30 years has led to a reduction of more than 50% for several of the most common disease issues in dairy cows (46). As a result, Norway's antibiotic usage per unit biomass is among the lowest for animal agriculture in the world (47).

In particular, the number of mastitis treatments has decreased markedly, with a 60% reduction achieved between 1994 and 2007. During the same period, bulk milk SCC more than halved (15). The trend has continued, with further decreases in the geometric mean somatic cell count in bulk tank milk and reported incidences of clinical mastitis occurring between 2009 and 2020 (21). These improvements have been attributed to accurate record keeping, improved breeding, increased preventive work (including disease specific control programs) and changing attitudes (15, 46). Despite these improvements, the national average score for the somatic cell count measure applied was still classified as problematic by WQ^®^.

The mean herd prevalence of ocular discharge, another disease-related measure, surpassed the alarm threshold. Ocular discharge is used in combination with nasal discharge, coughing, and hampered respiration to assess the respiratory health of the herd (10). The mean value for nasal discharge surpassed the WQ^®^ warning threshold but the mean scores for coughing and hampered respiration were well below their respective warning thresholds. The distinctly seasonal pattern of the prevalences of ocular and nasal discharge suggests that they may be the result of seasonal allergic rhinitis rather than viral or bacterial respiratory illnesses. Although seasonal allergic rhinitis has been little studied in cattle it has been reported (48, 49). This seasonal pattern was seen in a previous study of dairy cows using the WQ^®^ protocol (38).

It is clear that these potential clinical signs of disease, as assessed by the WQ^®^ protocol, remain a challenge in Norwegian dairy herds. The Norwegian dairy industry can take encouragement from the improvements they have made but they cannot afford to become complacent. Sustained efforts are required to maintain and improve the health status of Norwegian dairy cattle and thereby their welfare. It is important to differentiate between the clinical measures used to assess disease in the WQ^®^ protocol, which assess the clinical signs of disease, and confirmed instances of disease. If, as we suspect, the high prevalence of ocular discharge is caused by irritating seasonal rhinitis rather than potentially fatal pathogens then it is necessary to interpret that measure with caution. Similarly, as SCC is a measure of both sub-clinical and clinical mastitis, the welfare implications of the SCC results are not fully clear. Furthermore, as mentioned previously, the WQ^®^ assessment has been found to be poorly sensitive for improvements in these disease-related measures (36) and this aspect of the WQ^®^ protocol is under review.

Along with the criterion absence of disease, the criterion absence of injuries was low scoring with respect to the national average. Injuries are of concern for cow welfare as they are associated with pain. Lameness appears to be decreasing in loose-housed Norwegian dairy herds (16) but unfortunately the same cannot be said of integument alterations. Changes to the integument, most commonly of the tarsal, carpal, and neck regions were the most frequently encountered welfare issue in this study with almost four out of every five individuals affected on average. Alarmingly, the prevalence of integument alterations has remained stable over the past 13 years. In 2009, the prevalence of integument alterations was reported as 60.5% and 35.3% for the tarsal and carpal areas, respectively (14). We observed prevalences of 59.3 and 35.6% for the same issues using a similar scoring system. This suggests that they remain a challenge in Norwegian loose-housed dairy herds. The newly established National Animal Welfare Program for Cattle (50) includes a measurement of integument alterations, indicating that awareness of the issue is increasing and that improvement may be forthcoming. Prior to this the only national requirement related to the issue was a vague legislative requirement for dairy cows to have access to a soft, solid lying area (19). Previous research in Norway suggests that this requirement was not always met and failure to do so was a risk factor for integument alterations (14).

Moreover, despite rubber cubicle mattresses being considered “soft”, integument alterations of the tarsus have been found to be more prevalent, numerous, and severe in herds using mattresses (51). Rubber cubicle mattresses were used in all 155 herds. Additionally, skin alterations are an indicator of dysfunctional housing (14). Discomfort caused by challenges in the environment are also represented by the measure “mean time to lie down”. This is an integrative, animal-based measure of the interaction between all cubicle characteristics and the body dimensions of the cows (52). It is therefore an indicator of the suitability of the cows' resting environment. Discouragingly, this is higher compared to other studies (10, 11). Furthermore, nearly one in every ten herds were classified as having a severe problem with collisions during lying down movements. Such findings are consistent with prior evidence that resting comfort is more of an issue in cubicle herds as poorly designed, inadequately adjusted, and uncomfortably bedded cubicles can restrain cows during lying down movements and cause injuries (11).

Another management factor associated with integument alterations is the number of days per year spent at pasture. More time spent inside has previously been linked to increased integument alternations (53, 54). In almost entirely pasture-based systems the prevalence of integument alterations is far lower. One study of pasture-based dairy cows in Australia recorded that 86% of herds had no cows with any hairless patches, compared with 0% in this study. The same study found that 56% of herds had no cows with observed lesions, compared with < 1% in this study (53).

There is a statutory requirement for cows to have pasture access for at least 8 weeks of the year (19). Based on the results of this study, this requirement is generally met. Despite this, it appears that Norwegian dairy cows spend less time outside than in other countries. For example, the median number of days at pasture per year in this study was little over half the median number of 299 days observed in France (11). Pasture access is limited by the prevailing weather conditions for much of the year and a lack of high-quality pasture in mountainous areas. It could be that despite having some pasture access, the duration is not long enough to reduce the prevalence of integument alterations. A study investigating daily grazing time as a risk factor for tarsal integument alterations found that reductions in the log-odds for lesions and swellings on the tarsus joint were only present in the group which spent the most time per day outside (54). It is possible that there is a type of dose-response relationship between duration of pasture access and the occurrence and severity of integument alterations. The case may be that the effective “dose” of days at pasture is not consistently met under Norwegian conditions.

### 4.4. Regional variation

We hypothesized that the welfare status may vary regionally. Regional variation, if present, would be an important consideration when attempting to investigate the welfare status of a large, geographically diverse country like Norway. A regionally variable welfare status would also have implications for the implementation of region-specific welfare interventions or advisory strategies. The relationship between region and welfare is complex because, as a variable, it is the summary of all of the different observed and unobserved characteristics of said region. We stratified the observed results using GLM to investigate if the sum of these regional factors can influence the welfare status of regions to the extent that they differ significantly from one another.

A significant regional difference was found between two regions for only one for the principles, A. Good housing. The variation, while significant, was small. The possibility for variation between Trøndelag and Vestlandet North exists in measures related to the criterion comfort around resting as this was the only criterion which could vary between the regions. The associated measures were those of the indoor environment: cleanliness and the interactions between the cows and their resting facilities. Variation in the indoor environment was one of our hypothesized sources of variation between regions prior to the data collection. Unfortunately, due to the complex nature of the issues measured and a lack of specific information about the housing in these regions, it is not possible at this point to identify the cause(s) of the variation between Trøndelag and Vestlandet North.

We found no regional variation within Norway for the other three WQ^®^ principles. Regardless of whether the typical feed composition varies regionally, as we supposed, or not, it appears to meet the cows' nutritive requirements in most cases. We also assumed that the number of days per year at pasture would vary from region to region, but this assumption appears to be incorrect based on these data. The lack of variation on a regional basis, with one borderline exception, runs counter to our initial belief that welfare status would vary markedly from region to region. It has previously been suggested that when cows are kept in similar systems, their welfare is broadly similar from country to country (11). In this study, all herds were cubicle housed for most of the year with pasture access in summer. It may be that the same applies when cows are keep in similar systems intra-nationally.

A general absence of regional variation highlights the importance of variation from herd to herd. As mentioned in the results section, variability was present throughout the WQ^®^ scores (Table 4) and the prevalences/measures of common welfare issues (Table 5). The principle good feeding and its contributory criteria and measures showed particular high variability. As the variations in this data are not generally present at a regional level, they must instead be at a herd level. The role of the stockperson has been identified as an important determinant of the welfare at a herd level (55, 56).

### 4.5. Other causes of variation

To investigate the presence or absence of regional variations we were required to build generalized linear models, as described. In doing so we identified a number of factors which interacted with potential region effects.

In a country as large as Norway there was a necessity for multiple assessors. The WQ^®^ protocol was designed with inter- and intra-observer reliability in mind (10), however limitations have been identified previously in both dairy and pig herds (57–59). Even though steps were taken during training to ensure the reliability of the assessors' readings, a significant assessor effect was detected during univariable screening for three of the four models. Inter-observer reliability can be increased by more intensive training procedures, as has been demonstrated by lameness scoring (60). Unfortunately, it was not feasible to extend the training due to the limitations on physical meetings in place during the critical training period for this study. Assessor effects could act as confounders for regional effects because the visits performed by each assessor were not evenly distributed across the five regions: Assessor was therefore included as a fixed effect in all four models (A-D).

Herd size has been investigated previously for its effect on welfare parameters (38, 53, 61). A statistically significant positive relationship between herd size and the principle score for good housing was detected during univariable screening. In the authors' experience, this positive association is biologically plausible under Norwegian conditions as larger herds are more likely to be housed in more modern housing facilities. Newer housing solutions are conceivably more appropriately designed for modern dairy cows than older housing designs. Cows in larger herds may therefore experience more comfortable conditions, as demonstrated in the data by the positive linear association between herd size and principle score for good housing. Additionally, a curvilinear relationship between herd size and two measures which contribute to the good housing score has been shown in previous research on loose-housed dairy cows (38). Herd size was therefore included as an independent variable in model C. Good housing.

By including a binary variable indicating either passive or active recruitment during the univariable screening, we detected a significant positive effect of being passively recruited on the principles C. Good health and D. Appropriate behavior. In short, those who proactively volunteered their participation appeared to score significantly higher, on average, for those two principles (while also accounting for other factors such as region and assessor). Recruitment method was therefore retained in the best fitted models for those principles in order to account for the participation bias potentially identified.

## 5. Conclusion

Our assessments using the WQ^®^ protocol provided an approach to reporting the national status of animal welfare in loose-housed Norwegian dairy herds. The status both presented encouraging findings and identified welfare challenges. Particularly positive were the low herd prevalences of very lean cows and the positive relationship between stockpeople and their cattle. The challenges of most concern were injuries arising from conflicts between the cows and their environments, clinical signs such as ocular discharge, and inadequate resting comfort.

Our investigation revealed significant regional variation between just two regions in only one of the four principle scores assessed. The suggestion is that contrary to our initial belief, the welfare status at WQ^®^ principle level in Norwegian loose-housed herds did not vary on a regional basis in most cases. However, variations in dairy cow welfare were clearly present at a herd level. We conclude, therefore, that attempts to improve dairy cow welfare should be directed at individual herds rather than at a regional level.

The welfare of dairy cows is multidimensional and positive achievements in some respects may not compensate for deficiencies in other areas. Stakeholders in Norwegian dairy production should focus on herd-specific opportunities to improve animal welfare.

## Data availability statement

The datasets presented in this article are not readily available because of the need to maintain participant anonymity. Requests to access the datasets should be directed to CB, conor.barry@nmbu.no.

## Ethics statement

Animal ethics approval was not required as this was an observational study which did not influence the animal's normal way of life or cause anything other than an entirely temporary light pain or discomfort. This is in line with Section 4.7 of the Norwegian University of Life Science's Ethical Guidelines. Written informed consent was obtained from the owners of the participating herds for the participation of their animals in this study. This study complied with the requirements for data protection and written, informed consent in studies involving human participants described in Section 4.6 of the Norwegian University of Life Sciences' Ethical Guidelines. Written informed consent was obtained from all human participants for their participation in this study. Independent evaluation by the Regional Committee for Medical and Health Research Ethics was considered to not be applicable due to the absence of ethical concerns regarding human participation in this study.

## Author contributions

Conceptualization, study planning, and writing—review and editing: CB, KE-D, RG, SG, CW, and CK. Assessor training: CW. Data collection: CB, KE-D, RG, SG, and CK. Software, data analysis, visualization, and writing—original draft: CB, KE-D, and CK. All authors contributed to the article and approved the submitted version.
