# Corrigendum: Obtaining an animal welfare status in Norwegian dairy herds—a mountain to climb

**DOI:** 10.3389/fvets.2023.1213749

**Published:** 2023-05-30

**Authors:** Conor Barry, Kristian Ellingsen-Dalskau, Randi Therese Garmo, Stine Grønmo Kischel, Christoph Winckler, Camilla Kielland

**Affiliations:** ^1^Department of Production Animal Clinical Sciences, Faculty of Veterinary Medicine, Norwegian University of Life Sciences, Ås, Norway; ^2^Department for Animal Health, Animal Welfare and Food Safety, Norwegian Veterinary Institute, Ås, Norway; ^3^TINE SA, Oslo, Norway; ^4^Department of Sustainable Agricultural Systems, Institute of Livestock Sciences, University of Natural Resources and Life Sciences, Vienna, Austria

**Keywords:** animal welfare, Welfare Quality^®^, dairy cattle, loose-housed, regional variation, Scandinavia, prevalence, free-stall housing

In the published article, there was an error. An ethics statement was not included.

A correction has been made to the Ethics statements.

The corrected Ethics statements appear below:

“Animal ethics approval was not required as this was an observational study which did not influence the animal's normal way of life or cause anything other than an entirely temporary light pain or discomfort. This is in line with Section 4.7 of the Norwegian University of Life Science's Ethical Guidelines. Written informed consent was obtained from the owners of the participating herds for the participation of their animals in this study. This study complied with the requirements for data protection and written, informed consent in studies involving human participants described in Section 4.6 of the Norwegian University of Life Sciences' Ethical Guidelines. Written informed consent was obtained from all human participants for their participation in this study. Independent evaluation by the Regional Committee for Medical and Health Research Ethics was considered to not be applicable due to the absence of ethical concerns regarding human participation in this study.”

The authors apologize for this error and state that this does not change the scientific conclusions of the article in any way. The original article has been updated.

